# Absence of genetic isolation across highly fragmented landscape in the ant *Temnothorax nigriceps*

**DOI:** 10.1186/s12862-022-02044-3

**Published:** 2022-07-15

**Authors:** Marion Cordonnier, Dominik Felten, Andreas Trindl, Jürgen Heinze, Abel Bernadou

**Affiliations:** Lehrstuhl Für Zoologie/Evolutionsbiologie, University of Regensburg, Regensburg, Germany

**Keywords:** Genetic isolation, Gene flow, Landscape fragmentation, Spatial structure, *Temnothorax*, Relatedness

## Abstract

**Background:**

Human activities, including changes in agricultural landscapes, often impact biodiversity through habitat fragmentation. This potentially reduces genetic exchange between previously connected populations. Using a combination of nuclear and mitochondrial markers, we investigated (i) genetic diversity and population structure at multiple spatial scales and (ii) colony genetic structure and queen mating frequency in the ant species *Temnothorax nigriceps* in a highly anthropized environment.

**Results:**

Although the results highlighted genetic structure on a European spatial scale, they did not reveal an impact of fragmentation on a regional scale, and we did not observe any genetic population structure on a regional scale. Across all populations, regardless of their geographical location, colony structure suggested monogyny (a single queen per colony) and monandry (single mating). However, nestmates were more related than expected, indicating that large-scale dispersal does not fully prevent genetic isolation.

**Conclusions:**

Despite living in fragmented patches of habitat, populations of *Temnothorax nigriceps* are apparently genetically not isolated at a regional scale. However, large-scale dispersal alone does not prevent genetic isolation. The ecological requirements of *T. nigriceps* may explain their resilience to habitat fragmentation by allowing them to survive in very small patches of suitable habitat. The deeper investigation of the diversity of functional habitats for this species should allow to appreciate better the mechanisms permitting this species to overcome the negative impacts of fragmentation.

**Supplementary Information:**

The online version contains supplementary material available at 10.1186/s12862-022-02044-3.

## Background

Human activities convert natural habitats into a highly modified landscape, which is characterized by a high density of built-up areas and impervious surfaces and an intensification of agricultural activities in urban peripheries [1–3]. In Europe, the intensification of agriculture over the last 50 years has led to the simplification of agroecosystems through the decline in landscape heterogeneity, eroding the quantity and quality of habitat for a wide diversity of species [4, 5]. These landscape changes often negatively impact biodiversity by destroying favorable habitat, decreasing habitat quality, increasing fragmentation of the remaining habitat and increasing exposure to habitat edge effects [6, 7]. Examining the genetic structure of populations in such disturbed habitats therefore can give information about their functional connectivity, corresponding to individual movements within and among (sub-)populations, and allows to determine their long-term viability.

The fragmentation and loss of habitat not only affect rare species but also species that are widely distributed in fragmented landscapes and whose populations can have very small effective sizes [9]. Social insects are among the most diverse and ecologically important organisms on earth and comprise 75% of the world's insect biomass [10]. In social Hymenoptera, such as ants, ecological conditions and habitat characteristics not only influence the genetic structure of natural populations, but also affect many aspects of social behavior and the social and genetic organization of their colonies [11–13]. For instance, Heinze (1993) demonstrated that in *Leptothorax* sp. A, patchy habitats are associated with a higher proportion of multi-queen colonies and the predominance of non-dispersing, wingless queens [11]. In *Temnothorax longispinosus*, the availability of empty nest sites influences the number of queens per nest and the number of nests inhabited by individual colonies [14]. Hence, the genetic consequences of habitat fragmentation for ant species can range from the population scale to the colony scale.

*Temnothorax nigriceps* (Mayr 1855) nests predominantly in cracks and crevices in limestone rocks [15]. It is therefore restricted to patchily distributed habitats in rocky-calcareous grasslands, including rocky outcrops in the Franconian Jura, and thus appears to be suitable for the study of population structure. Calcareous grasslands in the Franconian Jura mountains near Kallmünz have existed at least since the Bronze Age. Their area has been significantly reduced over the second half of the 20^th^ due to the cessation of grazing or afforestation, and large areas were replaced by new grasslands, exempt of the rocky outcrops favorable for the studied species [16, 17]. As a result, Franconian Jura mountains, although historically constituting a highly suitable area for *T. nigriceps,* represent now a highly fragmented landscape for this species.

At present, nothing is known about the dispersal capacity of *T. nigriceps* and its response to fragmentation, neither on the scale of populations nor that of colonies. The objective of this study is therefore to investigate the genetic exchange dynamics in *T. nigriceps* in the Franconian Jura, in a landscape experiencing a strong decrease in the favorable habitats for this species. Because of the scale dependence of the ecological patterns related to fragmentation, changing the observation scale across a landscape may reveal different patterns, and the same phenomenon may occur differently at different scales [18, 19]. Such an impact of multiple scales is particularly relevant for small animals, such as ants, as less mobile species should respond to landscape patterns at finer spatial scales than more mobile taxa [20]. We therefore aim to describe and quantify the genetic structure of populations and colonies of *T. nigriceps* at different spatial scales, from a local to a biogeographic level, to understand if the scarcity of favorable habitats influences the genetic structure of this species and how habitat isolation affects colony composition and genetic colony structure. We hypothesize that because of the scarcity of suitable nest sites for this species, it should exhibit a strong genetic structure, both at a large European scale but also at a regional scale (i.e., in the Franconian Jura area). At a local (habitat) scale, we predict that the flight ability of sexuals of this species should preserve the genetic exchange among populations.

## Results

**Fig. 1:**
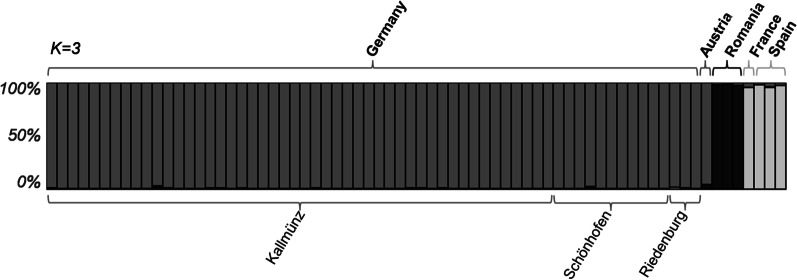
Structure barplot. Each individual is represented by a vertical line, which is partitioned into K colored segments that represent each individual's estimated membership fractions in K clusters (Q-values) from the consensus solution of the majority mode for the K = 3 Bayesian clustering assignment with a location prior. The locations on the top correspond to the locations incorporated to the model. The locations at the bottom indicate the individuals belonging to the three German sites

To determine if *T. nigriceps* exhibits population structure at the European scale, we collected 70 colonies from 5 European countries (Germany, Romania, Spain, Austria and France), separated by 60 to 2480 km from each other. Bayesian clustering analysis based on microsatellite genetic data at 12 loci without location prior did not reveal a significant genetic structure (only one homogeneous genetic cluster, Prob(K = 1) > 0.99). Investigation of structuring with location prior suggested three distinct genetic populations (Delta(K = 3) = 46.05; Prob(K = 3) > 0.99). All German samples were grouped in the same genetic cluster with the sample from Austria, whereas the other samples were divided into two clusters: the Romanian samples (N = 3) and the remaining 4 samples from France (N = 1) and Spain (N = 3; Fig. 1). The investigation of sub-structuring within the cluster of German and Austrian samples with the different German populations as location prior did not reveal a genetic structure (only one homogeneous genetic cluster, Prob(K = 1) > 0.98), suggesting no genetic differentiation among the three German sites.

These results are confirmed by the PCoA approach (Additional file 1: Fig. S1), although the Austrian sample clusters with the European samples instead of with the German ones along the first axis. F_ST_ values at the European scale also suggest a genetic differentiation between the different locations, with German samples genetically close to samples from Austria (F_ST_ = 0.1; Table 1A), and a very low genetic differentiation among the three German sites (F_ST_ ≤ 0.05; Table 1B).

**Table 1:** Pairwise F_ST_ values for A the five European locations and B the three German geographic sites

A	German sites	Kleinwalsertal, Austria	Aulus-les-Bains, France	Rimetea, Romania
Kleinwalsertal, Austria	0.10			
Aulus-les-Bains, France	0.12	0.63		
Rimetea, Romania	0.14	0.10	0.24	
Lérida, Spain	0.15	0.32	0.18	0.25

B	Kallmünz, Germany	Riedenburg, Germany		
Riedenburg, Germany	0.02			
Schönhofen, Germany	0.02	0.05		

**Fig. 2:**
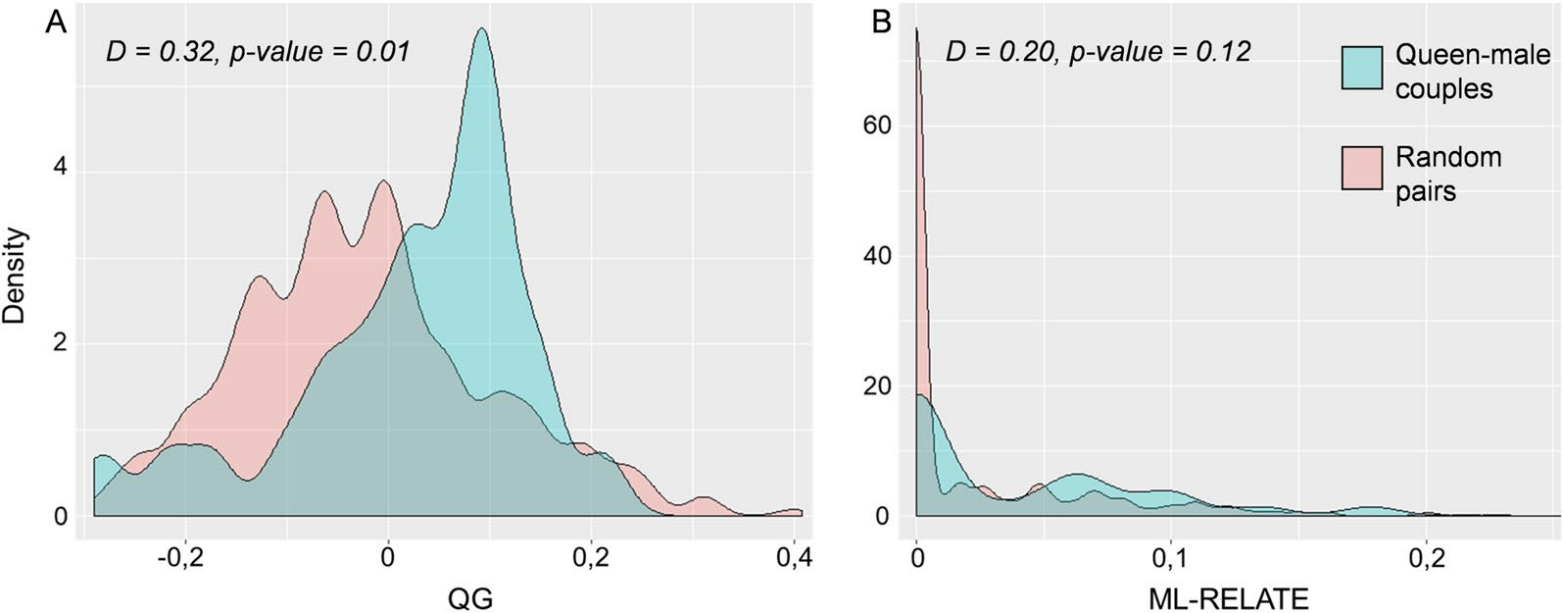
Pairwise relatedness between inferred queens’ and males’ genotypes based on **A** the estimator of Queller and Goodnight and **B** the maximum likelihood estimate ML-RELATE. Blue: relatedness between queen and males inferred from the same colony (n = 26); Red: relatedness between random pairs of queens and males (n = 650). Distribution comparison: nonparametric two-sample Kolmogorov–Smirnov test with one-sided alternative hypothesis

Colony sizes varied from 1 to 209 workers, with an average 104.3 ± 55.6 workers in Kallmünz and 112.3 ± 32 in Schönhofen, n = 63 colonies; however, the average increased to 118.9 ± 56.2 workers in Kallmünz and 130.2 ± 24.4 in Schönhofen based on n = 48 colonies, excluding those with fewer than 10 workers, which might have been incompletely collected (Additional file 1: Table S1).A single queen was found in 80% of the colonies (51/63), whereas no queen was collected in 17% of the colonies (11/63; Additional file 1: Table S1). Only one colony had two queens. In 29 of the 32 colonies, in which queen mating frequency was investigated, worker genotypes matched the hypothesis that a single male had mated with the queen (mean nestmate relatedness r = 0.77 ± 0.06 not different from the average relatedness r = 0.75 among sisters in haplodiploid species, single-sample t-test, t = 0.333, p = 0.741). In the three other colonies, the worker genotypes did not match the assumption of a single queen mated to only one male. Thus, we genotyped eight additional workers to increase reliability. In colony Tni38 (Kallmünz population, Germany), the workers resulted from one queen mated with two males, with two workers being offspring of the second male (r = 0.61). In Tni33 (Kallmünz population, Germany) and TniAu1 (Austria), only one of the 16 studied workers resulted from a different mother and father, suggesting the collection of a forager from another nest during sampling (Tni38: r = 0.66/r = 0.74; TniAu1: r = 0.68/r = 0.79 resp. with and without the alien worker). The calculations for pairwise relatedness between queens and males suggested different results depending on the estimator used. Based on the QG estimator, the effective paired couples were more related than expected regarding the pool of available potential mates (Kolmogorov–Smirnov test: D = 0.32, p-value = 0.01; Fig. 2A). The ML-RELATE estimator suggested a similar, although not significant trend (D = 0.20, p-value = 0.12; Fig. 2B). This signal of non-random mating translated not into inbreeding at the German population scale, as Fis = -0.06 was obtained within the German population (resp. Fis = 0,03 in Kallmünz, Fis = -0.16 in Riedenburg, Fis = -0.07 in Schönhofen), suggesting that the populations are at Hardy–Weinberg equilibrium.

**Fig. 3:**
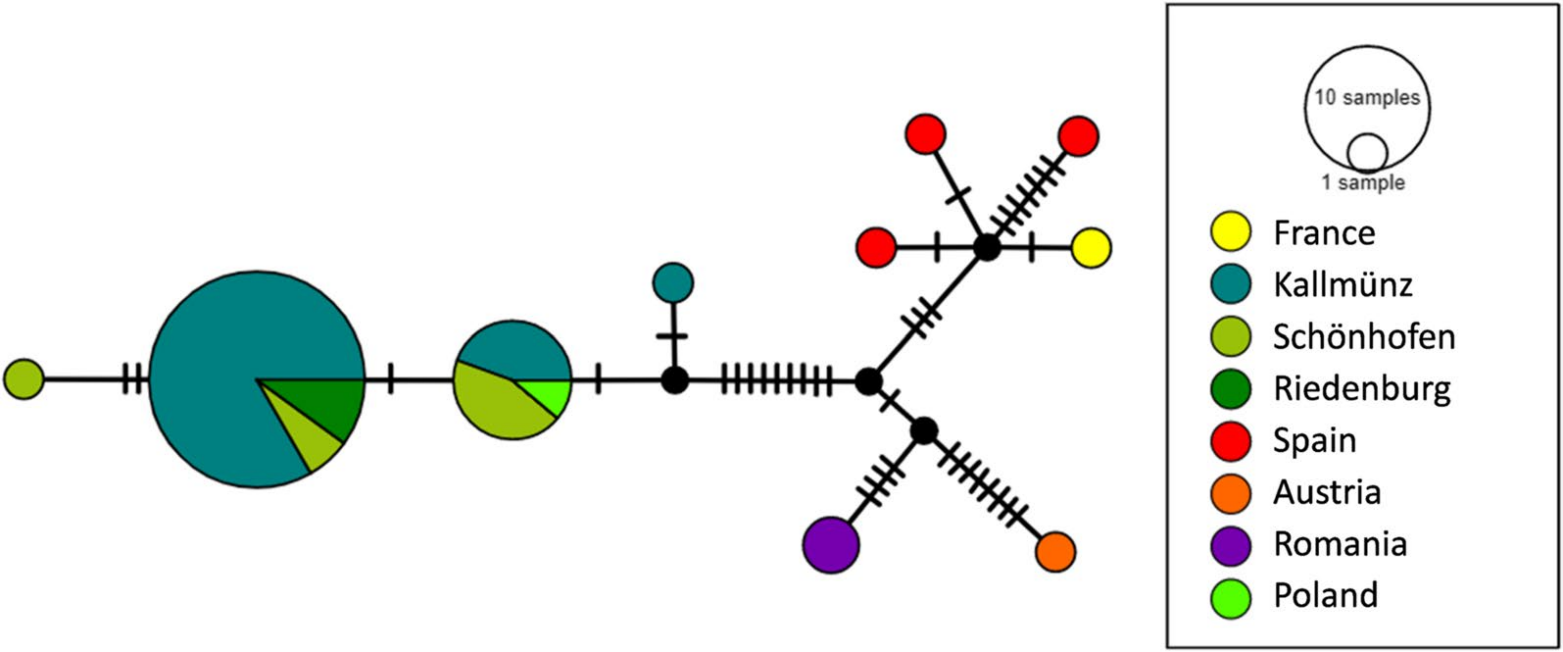
Haplotype networks based on the mitochondrial sequences. Each disk represents a haplotype. Disk surface is proportional to the number of individuals. The color of the disk indicates the geographical origin of the haplotype. The number of hatch marks corresponds to the number of mutations between two haplotypes

The PhyML tree based on the combined COI and COII sequences (See Additional file 1: Fig. S2) and the related haplotype network (Fig. 3) revealed a genetic structuring of the samples relating to their location at a large spatial scale, with haplotypes differing according to the sampling countries. French and Spanish samples shared similar haplotypes. However, at a finer spatial scale, the structuring did not correlate with the spatial structure of the sampling locations as the haplotypes from the three German populations were spatially mixed between the three sites (Fig. 3; Additional file 1: Fig. S2) as well as within the German site of Kallmünz (See Additional file 1: Fig. S3).

## Discussion

In this study, we investigated the impact of habitat scarcity on gene flow and genetic structure of colonies of the ant *T. nigriceps*. Although both nuclear and mitochondrial DNA suggested that distinct populations occur at a large European scale, no evidence of population genetic structure was detected at a regional or local scale. The results confirmed that the species has a monogynous, monandrous social colony structure across Europe. Within the German population, the paired mates were more related than expected under a random mating scenario when calculating relatedness using the Queller and Goodnight (1989) estimator.

Various processes acting both at large and fine spatial scales can drive the genetic structure observed. Although gene flow within ant species is not always constrained by large geographical barriers (see e.g., [21]), the genetic structure observable in the European populations of *T. nigriceps* both based on mitochondrial and nuclear DNA could be explained by a combination of isolation by distance and/or historical processes (e.g., restriction to refugia over ice ages). However, at smaller spatial scales, such as the regional scale investigated in the present study, these historical processes can be confounded with more recent disturbance on contemporary genetic admixture [22], such as habitat fragmentation, whether caused by the natural landscape (e.g., rivers; [23]) or by man-made structures (e.g., roads and urban areas; [24]). By inducing a disruption of dispersal processes, this fragmentation can threaten species that require specific habitats by causing the separation of populations whose effective sizes are not large enough to remain viable. Regarding *T. nigriceps* populations, the restricted habitats of this species combined with the presence of various potential physical barriers to dispersion (agricultural areas, impervious surfaces, rivers…) were therefore assumed to induce a strong genetic structure of the species at the regional scale around Regensburg. The results of the present study indicated a lack of genetic differentiation between German populations of *T. nigriceps* separated by more than 15 km. This could suggest that the small queens of this ant have surprisingly large dispersal abilities. In ant species with long-range dispersal strategies, gynes typically mate during large flights away from the nests and often found monogynous colonies [25–27]. Similarly, gynes of *T. nigriceps* disperse by flight [15] and colonies are monogynous, suggesting that this species also has high dispersal abilities and that gene flow can overcome local fragmentation between the remnant patches of suitable habitats. However, it remains unlikely that dispersal abilities alone allow the species to cross large patches of unsuitable habitat (e.g., recent agricultural landscape) to connect populations separated by more than 15 km. Diffusion by nuptial flight might therefore not be the only mode of dispersal. For example, *T. nigriceps* might be able to use man-made local microhabitats, such as stone walls [15] or other rocky elements of urban settlements (large urban parks infrastructures, cemeteries…) as stepping stones. Utilization of such artificial structures indeed provides wildlife, including ants [19] with alternative forms of shelter in fragmented environments [28].

Similarly, the related species *Temnothorax nylanderi* displays only weak population genetic structure in urban habitats, probably due to the micro-geographical home ranges of this forest species [29]. Nevertheless, these results remain surprising as fragmentation frequently induces genetic differentiation in ants (see e.g., [30, 31]), including in the closely related species *Temnothorax crassispinus* (J. Giehr, J. Heinze, unpublished data).

At the finer scale studied (colony structure within populations), *T. nigriceps* exhibited non-random mating as mating pairs were more related than pairs of sexuals randomly drawn from the population. As a locally limited dispersal is unlikely in species with nuptial flights (see above), this pattern was unexpected and is difficult to explain. A synchronic initiation of nuptial flights in nests sharing micro-environmental conditions could explicate this pattern: more distant nests, experiencing different climatic conditions, might produce sexuals in different short periods of summer, leading to local nuptial flights. Such local, asynchronous mating has been observed in several ant species (e.g., *Messor aciculatus*, [32]) including *T. nylanderi*, where mating probably occurs during several small, local nuptial flights involving only sexuals from few, presumably neighboring colonies [33]. This might result in an increased relatedness among mating pairs relative to sexuals from the whole population. The lack of genetic differentiation in mtDNA across collecting sites in Germany indicates that after mating queens disperse further away. Male dispersal might be more limited due to their smaller size. In *Temnothorax longispinosus*, the smallest gynes exhibit a reduced flight activity that may encourage returning to the natal nest after mating, suggesting that mating swarms are not distant from the natal nest in this species [34]. Nuptial flights occurring close to the natal nest, at least regarding the males’ dispersal, could also produce the patterns observed in *T. nigriceps*.

Our study confirmed the monogyny of *T. nigriceps* and did not give evidence of the fusion of colonies or the adoption of alien queens or workers as observed in *T. nylanderi* and *T. crassispinus*. This reflects the different nest sites inhabited by the species. Nest sites of *T. nigriceps*, e.g., rock crevices, are comparatively stable compared to those of *Temnothorax* species nesting in decaying branches and hollow acorns (e.g., [35, 36]), which could partly prevent colony fusion. Nevertheless, the availability of nest sites suitable for *T. nigriceps* is likely restricted, with few crevices locally available. This might suggest high competition for appropriate nests. A deeper investigation of local colony densities, for instance regarding large boulders as a patch of accessible habitat for colony relocation, together with an experimental approach investigating the competitive behavior between colonies, could allow to decipher the conflict resolution in this species. Finally, among the 32 colonies investigated, only one occurrence of polyandry has been detected, suggesting either that queens are generally singly inseminated and polyandry occurs very rarely in the species, or that our method was not accurate enough to detect very unequal contributions of multiple males to the offspring of queens. In the colony displaying polyandry, only 12% of the workers were sired by the second male, suggesting that strong biases can occur toward one of the mated partners. In such a situation, the number of workers investigated in each colony could be insufficient to detect polyandry [37], inducing a possible underrepresentation of multiple mating in the present study.

## Conclusions

Despite living in very specific remnants of habitat that have been largely replaced by less favorable grasslands in the last century, constituting for the ant *T. nigriceps* a highly fragmented landscape, populations of this species are apparently genetically not isolated at a regional scale. The results observed at the European scale suggest that the current genetic structure of the species results from past historical events rather than recent fragmentation. The investigation of genetic structuring at intermediate spatial scales would allow to untangle the drivers of gene flow within this species and to investigate demographical events implied. Models studying genetic structure at a landscape scale could be less appropriate for small species exploiting micro-habitats such as ant species, as their local ecological and/or life-history requirements drive species-specific patterns of landscape functional connectivity [19, 29]. The incorporation of the micro-habitat scaled mechanisms would benefit further studies aiming to understand the drivers of *T. nigriceps* population structure within landscapes and genetic exchanges between colonies at a local scale. The deeper investigation of ecological requirements as well as the diversity of functional habitats for this species should allow to appreciate better the mechanisms permitting this species to overcome the negative impacts of habitat fragmentation.

## Methods

### Sampling

**Fig. 4:**
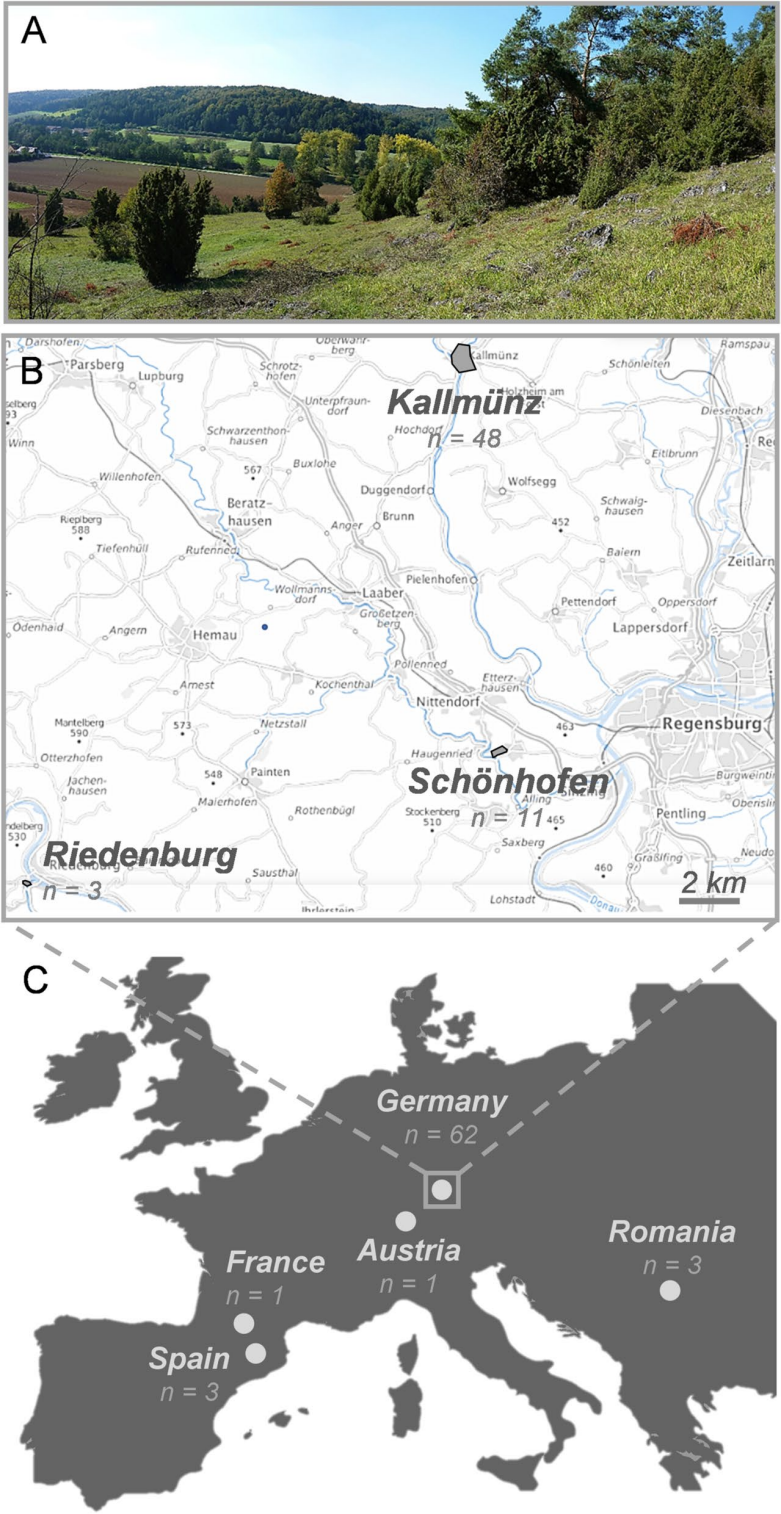
Map of the sampling areas. **A** Picture of one of the sampling locations in Kallmünz; sampling habitats correspond to the foreground natural rocky area © Abel Bernadou. **B** German sampling sites © Bundesamt für Kartographie und Geodäsie (2020). **C** European sampling scale

*Temnothorax* is a diverse genus occurring in most parts of the world, with more than 150 species in Europe, Caucasia, and Anatolia alone [15]. Colonies are usually small (< 200 workers; [15, 38]) and occasionally distributed among satellite nests (e.g., [31, 37–40]). T. nig*riceps* (Mayr 1855) is a European ant species living in sunny xerothermous habitats with sparse vegetation and bare rock (Fig. 4A). Colonies nest in narrow crevices in solid rock or in the clefts of stone walls [15]. According to Seifert (2018), *T. nigriceps* is a strictly monogynous species and the female sexuals (gynes) disperse by flight for mating and claustral colony founding [15]. Although *T. nigriceps* inhabits rare, fragmented habitats and might therefore be of interest for research on gene flow in anthropized landscapes, dispersal and mating tactics have not yet been investigated in more detail and most previous studies only mention its occurrence in different European locations [43–45].

In this study, 70 T*. nigriceps* colonies separated by at least 5 m from each other were collected between June and September in 2011, 2012, and 2021 and stored in 90% ethanol. Among them, 62 colonies were collected in three distinct sites near Regensburg, Germany: Kallmünz (48 nests collected in 2011–2012 in a large rocky-calcareous grassland of around 3.5 km^2^), Schönhofen (7 nests collected in 2011–2012 and 4 nests collected in 2021 in a medium-size rocky-calcareous grassland patch of 73,600 m^2^) and Riedenburg (3 nests collected in 2021 in a small patch of suitable habitat of 2300 m^2^ near a ruined castle) (Fig. 4B). Additionally, eight colonies were collected in other European regions (Rimetea, Romania, 3 nests; Aulus-les-Bains, France, 1 nest; Lérida, Spain, 3 nests; Riezlern, Kleinwalsertal, Austria, 1 nest, Fig. 4C). Whenever possible, the whole colonies were collected by opening or cracking the crevices occupied by the individuals. Apart from the samples of 2021, the number of queens and workers have been recorded for each colony (see Additional file 1: Table S1 for details).

### Molecular analyses

DNA was extracted from one worker in each of the 70 colonies. For 32 of the 70 colonies, DNA from seven additional workers was extracted (23 colonies from Kallmünz, 3 from Schönhofen, 1 from Austria, 3 from Romania, 2 from Spain), and for 10 of these colonies we also extracted the queen (see Additional file 1: Table S1 for details). In case of irregular observations in the colony structure (see below), DNA from eight additional workers were extracted. DNA was extracted using a CTAB method (modified from [46]).

#### Microsatellite data

Twelve highly variable microsatellite markers were used to determine the genetic population and colony structure: LX GT 218 [47], Ant3993 and Ant11893 [48], L-18 [49], LXA GT 1 [50], 2MS17, 2MS46, 2MS60, 2MS67, 2MS82, 2MS87, and 2MS91 [51]. Primer sequences are available in Additional file 1: Table S2. For all markers except Ant11893, the 10 µl PCR reaction volume consisted of 5 µl buffer with Taq DNA polymerase, 3 µl ddH2O, 0.5 µl unlabeled reverse primer, 0.5 µl labelled forward primer (HEX, FAM and TET; final concentration of 0.5 µM) and 1 µl DNA (2–10 ng). For Ant11893, the 15 µl PCR reaction volume consisted of 7.5 µl buffer with Taq DNA polymerase, 5 µl ddH2O, 1 µl unlabeled reverse primer, 1 µl labelled forward primer (HEX, FAM and TET; final concentration of 0.5 µM) and 0.5 µl DNA (2–10 ng). For LX GT 218, Ant3993, L-18 and LXA GT 1, PCR consisted of initial denaturation at 94 °C (4 min), 33 cycles at 94 °C (denaturation, 45 s), 57 °C (annealing, 80 s) and 72 °C (elongation, 25 s), and a final step at 72 °C (1 min). For 2MS17, 2MS46, 2MS60, 2MS67, 2MS82, 2MS87, 2MS91 and Ant11893, PCR consisted of initial denaturation at 94 °C (3 min), 33 cycles at 94 °C (denaturation, 45 s), 55 °C (annealing, 30 s) and 72 °C (elongation, 30 s), and a final step at 72 °C (5 min). The PCR products were analyzed in an ABI PRISM 310 Genetic Analyser (PE Biosystems) after DNA denaturation at 90 °C (1 min). Allele sizes were determined using genescan 3.1 software (PE Biosystems). In case of eventual failure of PCR or unclear results, the molecular analysis was repeated to ensure that genotypic information on at least 10 successful loci was obtained for all individuals at all loci. All twelve loci were polymorphic and showed considerable variation with an average of 19.7 alleles across all samples (allele numbers; LX GT 218: 12, Ant3993: 9, L-18: 28, LXA GT 1: 27, 2MS17: 27, 2MS46: 21, 2MS60: 26, 2MS67: 9, 2MS82: 20, 2MS87: 11, 2MS91: 26, Ant11893: 22). To ensure the quality of the markers, the number of alleles, expected and observed homozygotes, the frequency of null alleles and the presence of stuttering or large allele dropouts were controlled and the deviation to the Hardy Weinberg Equilibrium was tested at each locus using Micro-Checker [52] and Genepop v4.5.1 [53, 54]. Tests for linkage disequilibrium have been performed between all pairs of primers using Genepop v4.5.1 [51, 52]. The p-values were corrected for multiple tests (Holm correction; [55]) using the package *multcomp* [56] in R v. 4.0.2 [57] (Additional file 1: Tables S3 and S4).

#### Mitochondrial data

Mitochondrial sequences were obtained based on PCRs conducted on 47 individuals (30 colonies from Kallmünz, 3 from Riedenburg, 7 from Schönhofen, 1 from Austria, 2 from Romania, 3 from Spain, 1 from France; see Additional file 1: Table S1 for details). Two primer combinations were used: C1-J-2183 "Jerry"/Cw.3031 (resp. 5’-CAACATTTATTTTGATTTTTTGG-3´/5´-TTTGC(A/C)CT(A/T)ATCTGCC(A/C)TATT-3´) and COI-516for/COII “C2-N-3661” (resp. 5´-ATTTTT(T/C)TCTATATTTAT(T/C)GGA-3´/5´-CCACAAATTTCTGAACATTGACCA-3´; [58]). The 25 µl PCR reaction volume consisted of 12.5 µl buffer with Taq DNA polymerase, 9.5 µl ddH2O, 1 µl reverse primer, 1 µl forward primer (final concentration of 0.5 µM) and 1 µl DNA (2–10 ng). PCR consisted of initial denaturation at 94 °C (4 min), 37 cycles at 94 °C (denaturation, 45 s), 50 °C (annealing, 45 s) and 72 °C (elongation, 60 s), and a final step at 72 °C (5 min). PCR products were cleaned up using the NucleoSpin Gel and PCR Macherey–Nagel’s Clean-up Kit and sequencing was conducted by LCG Genomics GmbH (Berlin, Germany).

### Bayesian admixture model

To determine the number of genetically homogeneous groups using microsatellite data, we used the Bayesian clustering algorithm implemented in the software STRUCTURE v. 2.3.1 [59] based on the admixture model with correlated allele frequencies, and with the number of a priori unknown clusters (K) varying from K = 1 to 5 (i.e., the number of European sampling areas), with 10 iteration runs for each K-value. Each run consisted of 500,000 replicates of the MCMC after a burn-in of 500,000 replicates. The same model was used twice, with and without sampling area as prior location of samples [60]. To compare the 10 independent runs, clustering results were analyzed using CLUMPAK [61] based on a Markov clustering algorithm, which groups sets of highly similar runs into modes and separates these distinct groups of runs to generate a consensus solution for each distinct mode. For any given K, the different runs either generated a consensus solution with a single mode or resulted in both a majority mode consisting of most of the iterations and one or more minority modes consisting of the remaining iterations. CLUMPAK was then used to identify an optimal ordering of inferred clusters across different values of K, and to define the optimal K-value using the method of Evanno et al. [62]. The mean likelihood for each K according to Pritchard et al. [59] was calculated using Structure Harvester [63]. The most conservative value of K compatible with these different elements – concordance between runs, Evanno et al. method [62] and mean lnP(K) – was retained. The membership coefficient of each individual at each of the K clusters corresponding to the consensus solution of the majority mode was selected as Q-value. At each hierarchical level, individuals were grouped assuming a membership coefficient of at least 50% to belong to a cluster [64]. For clusters incorporating more than 10 individuals, the same process was then separately iterated hierarchically within each cluster to measure sub-structuring within the identified clusters. All parameters remained identical except for the maximum number of clusters tested (K), which systematically corresponded to the number of sampling areas involved in the hierarchical level (including the three collecting sites within the German cluster). We considered that a cluster was genetically homogeneous when no individuals in this cluster had a Q-value greater than 0.9 at the next hierarchical level.

### Genetic differentiation between sites

To confirm the results of the hierarchical clustering process, and given the fact that STRUCTURE is sensitive to unbalanced sampling [65], the genetic structure has been investigated through a Principal Coordinate Analysis (PCoA) using the software GenAlEx [66]. The degree of genetic differentiation at the population level was also estimated using the pairwise F_ST_ for (i) the five European locations and (ii) the three German geographic sites using Genepop. The degree of genetic differentiation was defined as low (0 ≤ F_ST_ < 0.05), medium (0.05 ≤ F_ST_ < 0.15), high (0.15 ≤ F_ST_ ≤ 0.25) or very high (F_ST_ > 0.25; [67]).

### Observed number of matings

Based on the eight worker genotypes of the 32 colonies studied (see Additional file 1: Table S1), we inferred the genotypes of queens and their mates in each colony. At each locus, two alleles shared by all the workers were assigned to the queen, while the putative genotypes of the haploid fathers were determined from the alleles unassigned to the mother. This pattern was iterated over the 12 markers until reaching a minimal number of queens and mates per queen. To ensure the robustness of the method, the relatedness between workers from the same nest was calculated using the estimator of Queller and Goodnight [68] provided by GenAlEx [66]. In addition, the queens of 10 colonies were genotyped and we systematically compared the empirically found genotypes of queens with those inferred from the genotypes of workers. Situations in which more than one potential queen/male genotype was inferred based on the genotypes of the eight workers (n = 3, Additional file 1: Table S1) motivated the genotyping of eight additional workers to ensure the consistency of the result within the colony.

### Genetic relationship between mates

Within the German population (i.e., the 23 colonies from Kallmünz and 3 colonies from Schönhofen), pairwise relatedness between the inferred genotypes of queens and males was calculated using two different estimators. The estimator of Queller and Goodnight [68] allowed to determine the genotypic similarity of microsatellite markers between pairs of individuals compared to an expected value between two individuals taken at random from the population. Negative values indicated that the degree of kinship between the two individuals tested was less than that of individuals drawn randomly from the population. Relatedness was also calculated using ML-RELATE [69], which evaluated maximum likelihood estimates of pairwise relatedness between individuals based on simulations to compare putative relationships with alternatives and generate absolute non-negative estimates. For both estimators (QG and ML-RELATE), the relatedness between pairs of queens and their mates (n = 26; Additional file 1: Table S1) was compared with the relatedness among random pairs of queens and males (n = 650 random pairs) using a nonparametric two-sample Kolmogorov–Smirnov test. The empirical cumulative distributions of the relatedness obtained were compared using a one-sided alternative hypothesis to decipher if paired couples were more related than expected considering the global relatedness between reproductives in the population.

### Haplotype analysis

Global relationships among the 47 mitochondrial haplotypes were based on consensus sequences of the COI and COII sequences, corresponding to fragments ranging from 1156 to 1400 bp lengths. The sequences generated in this study were compared with two sequences obtained from GenBank for two *Temnothorax* species (GenBank accession number: MF436633.1 for *T. nigriceps* – sample from Poland; MF436635.1 for *T. nylanderi* – sample from Spain). All sequences were aligned using the default options in MUSCLE v3.8.31 [70] as implemented in SeaView v4.2.9 [71]. The relationships were evaluated based on a tree constructed using the PhyML algorithm with the GTR distance without invariable sites, optimized nucleotide equilibrium frequencies, and tree-searching operations involving best of NNI & SPR. PopART v. 1.7 [72] was used to build a haplotype network using the Median Joining inference under default settings and applying a provided trait file coding for locality information of samples.

## Supplementary Information

**Additional file 1. **Additional tables and figures.

## Data Availability

The Genotype raw data generated during the current study are available in Zenodo repository [https://doi.org/10.5281/zenodo.6359883]. The COI sequences generated during the current study are available in Genebank repository [Accession Numbers ON052777-ON052823].
