# Supplementary material for: Absence of genetic isolation across highly fragmented landscape in the ant Temnothorax nigriceps
Source: BMC Ecol Evol. 2022 Jul 15;22:91. doi: 10.1186/s12862-022-02044-3 (PMC9284864; doi:10.1186/s12862-022-02044-3)

Additional file 1

Absence of genetic isolation across highly fragmented landscape in the ant *Temnothorax nigriceps*

Marion Cordonnier[a1], Dominik Felten[a], Andreas Trindl[a], Jürgen Heinze[a*], Abel Bernadou[a*]

[a]Lehrstuhl für Zoologie / Evolutionsbiologie, Univ. Regensburg

[*]Equal contribution

[1]Corresponding author – marion.cordonnier@hotmail.fr

Table S1. Information regarding the 70 colonies of *Temnothorax nigriceps* used in the study. *Coll. Date*: date of sampling. *Location*: Source population; Kallmünz, Riedenburg and Schönhofen correspond to the three German populations. Queens / Workers nb.: Number of queens / workers collected. *Coordinates DMS*: geographical coordinates of the collected nest. *Workers' genotypes nb.*: Number of workers genotyped; * : colony whose queen was genotyped; †: Colonies whose genotypes were used for relatedness analysis. *Sequencing*: list of samples used for the haplotypic analysis.

Nest ID	Coll. date	Location	Queens nb.	Workers nb.	Coordinates DMS		Workers' genotypes nb.	Sequencing
TNi Au1	11.07.2013	Riezlern, Kleinwalsertal	1	>10	47°23'15"N	10°13'15"E	16*	x
TNi FR1	12.06.2010	Aulus-les-Bains	0	3	42°48'11.5"N	1°21'39.5"E	1	x
TNi6	06.09.2011	Kallmünz	1	196	49°9'56.6"N	11°56'36.4"E	8*†	x
TNi7	06.09.2011	Kallmünz	1	24	49°9'57.0"N	11°56'36.8"E	8†	x
TNi2	26.08.2011	Kallmünz	1	164	49°10'5.5"N	11°57'19.8"E	8*†	x
TNi1	26.08.2011	Kallmünz	1	126	49°10'5.2"N	11°57'19.7"E	8†	x
TNi3	26.08.2011	Kallmünz	1	94	49°10'5.3"N	11°57'19.0"E	1	x
TNi12	06.09.2011	Kallmünz	1	65	49°10'3.4"N	11°57'20.1"E	1	x
TNi13	06.09.2011	Kallmünz	1	200	49°10'3.4"N	11°57'21.9"E	1	x
TNi40	03.10.2011	Kallmünz	1	209	49°09'36.8"N	11°58'00.4"E	8†	x
TNi41	03.10.2011	Kallmünz	1	66	49°09'36.5"N	11°58'01.1"E	8†	x
TNi38	03.10.2011	Kallmünz	1	181	49°09'37.5"N	11°57'57.8"E	16*†	x
TNi64	30.06.2012	Kallmünz	1	134	49°09'44.5"N	11°57'45.0"E	8†	x
TNi68	30.06.2012	Kallmünz	1	120	49°09'44.3"N	11°57'47.4"E	8†	x
TNi69	30.06.2012	Kallmünz	1	102	49°09'44.6"N	11°57'47.0"E	8†	x
TNi71	30.06.2012	Kallmünz	1	78	49°09'42.9"N	11°57'47.5"E	8†	x
TNi66	30.06.2012	Kallmünz	1	176	49°09'44.3"N	11°57'47.3"E	1	x
TNi19	06.09.2011	Kallmünz	1	188	49°10'0.4"N	11°58'30.9"E	8*†	x
TNi21	02.10.2011	Kallmünz	1	139	49°09'59.5"N	11°58'30.0"E	8†	x
TNi5	26.08.2011	Kallmünz	1	186	49°10'0.6"N	11°58'36.4"E	1	x
TNi22	02.10.2011	Kallmünz	1	83	49°10'00.3"N	11°58'27.5"E	1	x
TNi27	02.10.2011	Kallmünz	1	176	49°08'07.5"N	11°56'39.1"E	8*†	x
TNi55	11.10.2011	Kallmünz	1	176	49°08'07.7"N	11°56'38.9"E	8†	x
TNi58	11.10.2011	Kallmünz	1	22	49°08'08.1"N	11°56'39.5"E	8†	x
TNi59	11.10.2011	Kallmünz	1	119	49°08'07.7"N	11°56'39.3"E	8†	x
TNi28	03.10.2011	Kallmünz	1	154	49°09'01.5"N	11°57'21.7"E	8†	x
TNi36	03.10.2011	Kallmünz	1	138	49°09'01.7"N	11°57'25.9"E	8†	x
TNi33	03.10.2011	Kallmünz	1	146	49°09'01.1"N	11°57'23.8"E	16*†	x
TNi30	03.10.2011	Kallmünz	1	87	49°09'01.5"N	11°57'22.5"E	1	x
TNi31	03.10.2011	Kallmünz	1	198	49°09'01.3"N	11°57'22.8"E	1	x
TNi37	03.10.2011	Kallmünz	1	105	49°08'59.2"N	11°57'28.9"E	1	x
TNi42	03.10.2011	Kallmünz	1	193	49°09'05.9"N	11°57'18.0"E	1	x
TNi8	06.09.2011	Kallmünz	1	1	49°9'58.8"N	11°56'36.3"E	1	
TNi9	06.09.2011	Kallmünz	1	128	49°10'5.2"N	11°57'20.0"E	8†	
TNi4	26.08.2011	Kallmünz	0	44	49°10'5.3"N	11°57'19.1"E	1	
TNi15	06.09.2011	Kallmünz	0	97	49°10'4.3"N	11°57'25.7"E	1	
TNi72	30.06.2012	Kallmünz	1	86	49°09'37.6"N	11°57'56.5"E	8†	
TNi39	03.10.2011	Kallmünz	1	1	49°09'37.6"N	11°57'59.7"E	1	
TNi60	30.06.2012	Kallmünz	1	2	49°09'43.9"N	11°57'44.5"E	1	
TNi62	30.06.2012	Kallmünz	1	70	49°09'44.1"N	11°57'44.4"E	1	
TNi65	30.06.2012	Kallmünz	1	58	49°09'44.5"N	11°57'46.3"E	1	
TNi67	30.06.2012	Kallmünz	1	38	49°09'44.5"N	11°57'47.5"E	1	
TNi18	06.09.2011	Kallmünz	0	173	49°10'0.0"N	11°58'32.1"E	8†	
TNi16	06.09.2011	Kallmünz	1	1	49°9'59.6"N	11°58'37.0"E	1	
TNi17	06.09.2011	Kallmünz	0	15	49°10'0.7"N	11°58'35.9"E	1	
TNi23	02.10.2011	Kallmünz	0	53	49°10'00.9"N	11°58'27.6"E	1	
TNi24	02.10.2011	Kallmünz	1	2	49°10'00.8"N	11°58'28.5"E	1	
TNi20	02.10.2011	Kallmünz	1	112	49°08'08.5"N	11°56'37.5"E	1	
TNi26	02.10.2011	Kallmünz	1	5	49°08'07.4"N	11°56'39.0"E	1	
TNi35	03.10.2011	Kallmünz	0	75	49°09'01.0"N	11°57'24.8"E	1	

TNi21.11	11,06,2021	Riedenburg	NA	> 30	48°57'38.0"N	11°40'56.5"E	1	x
TNi21.12	11,06,2021	Riedenburg	NA	> 30	48°57'38.2"N	11°40'55.7"E	1	x
TNi21.14	11,06,2021	Riedenburg	NA	> 30	48°57'38.2"N	11°40'56.1"E	1	x
TNi R3	23.09.2011	Rimetea	1	192	46°25'37.89"N	23°32'37.83"E	8*	x
TNi R1	23.09.2011	Rimetea	1	161	46°25'38.23"N	23°32'38.17"E	8	x
TNi R6	18.09.2011	Rimetea	0	41	46°27'0.08"N	23°34'35.36"E	8	
TNi49	04.10.2011	Schönhofen	1	133	49°00'15.1"N	11°58'16.3"E	8*†	x
TNi46	04.10.2011	Schönhofen	1	173	49°00'14.7"N	11°58'17.6"E	8†	x
TNi54	04.10.2011	Schönhofen	1	139	49°00'13.4"N	11°58'16.5"E	8†	x
TNi21.1	11,06,2021	Schönhofen	NA	> 30	49°00'12.9"N	11°58'10.6"E	1	x
TNi21.2	11,06,2021	Schönhofen	NA	> 30	49°00'12.4"N	11°58'09.4"E	1	x
TNi21.4	11,06,2021	Schönhofen	NA	> 30	49°00'12.9"N	11°58'12.8"E	1	x
TNi21.5	11,06,2021	Schönhofen	NA	> 30	49°00'13.8"N	11°58'12.1"E	1	x
TNi43	04.10.2011	Schönhofen	2	5	49°00'15.3"N	11°58'16.9"E	1	
TNi44	04.10.2011	Schönhofen	0	117	49°00'14.5"N	11°58'16.8"E	1	
TNi45	04.10.2011	Schönhofen	0	106	49°00'14.4"N	11°58'16.6"E	1	
TNi48	04.10.2011	Schönhofen	0	113	49°00'15.0"N	11°58'16.1"E	1	
TNi SP1	07.2012	Lérida	1	>10	42°17'05.1"N	1°32'33"E	8*	x
TNi SP2	07.2012	Lérida	1	>10	42°17'05.1"N	1°32'33"E	1	x
TNi SP3	07.2012	Lérida	1	>10	42°17'05.1"N	1°32'33"E	8	x

Table S2. Microsatellite primers used in the study (Annealing temperature, orientation and sequence of primers).

	Annealing temperature [°C]	Orientation	Sequence of primers
LX GT218	57	Forward	5’-GTTCTTGCGCGGATGCATAC-3’
		Reverse	5’-TGTACTCGCGTGTCTATCGG-3’
Ant3993	57	Forward	5’-TGATCCGCTCTTAAAATTTAGATGGA-3’
		Reverse	5’-ACTTTCCGCRGCATTAAACATTTTCTT-3’
L-18	57	Forward	5’-TGAATTTGGATGGCGGTAGAC-3’
		Reverse	5’-ACCTAATGCACGCTTTAGAAT-3’
LXA GT1	57	Forward	5’-GTGGCGACCAATTCTGCAAG-3’
		Reverse	5’-GCAGGACCAGCATCAAATGACAG-3’
2MS17	55	Forward	5’-CAGCCTCTATTTTGTTCGAAG-3’
		Reverse	5’-TTTACTGCGGCTCCATAATC-3’
2MS46	55	Forward	5’-GCTCACTACTATGCTGCCAGC-3’
		Reverse	5’-CTTTCCTGCAAACCACGTGT-3’
2MS60	55	Forward	5’-TATGCGCCGGACAATAATCGC-3’
		Reverse	5’-GTTCATTGTCCGAGGCGCAGC-3’
2MS67	55	Forward	5’-GAAGATTCGTCAGGATGCAGC-3’
		Reverse	5’-AACTCTCGCTGGCAAGCGAGC-3’
2MS82	55	Forward	5’-AAAAGAGCATGCAACAGGTCAGC-3’
		Reverse	5’-TTTCTTAAGTCGCAAGCGAGC-3’
2MS87	55	Forward	5’-GGAACCTCACTCAACCTCGGT-3’
		Reverse	5’-ACGCGGACTACTTTAACCGGA-3’
2MS91	55	Forward	5’-AAAGTCTCGGAGTGGCTTTGC-3’
		Reverse	5’-ATTCTCGTCCATTTGTTCTAA-3’
Ant11893	55	Forward	5’-CAGGCTCGGRACGTTAATGC-3’
		Reverse	5’-GGTGCCGACGTCTAGCTAGC-3’

Table S3. Population genetics basic statistics. Number of alleles (Nb. Alleles), Observed and expected heterozygosity (Ho, He), presence of stuttering, dropout or null alleles, null allele frequencies, probability of Hardy Weinberg Equilibrium (HWE p-value) of each microsatellite marker.

	Nb. Alleles	He	Ho	stuttering	dropout	null alleles	null allele frequency	HWE p-value
LX GT 218	12	9.91	9	No	No	No	<0.01	> 0,99
Ant3993	9	11.82	17	No	No	No	0.21	0.03
L-18	28	3.81	9	No	No	yes	0.06	> 0,99
LXA GT1	25	5.64	11	No	No	yes	0.06	> 0,99
2MS17	27	4.40	8	No	No	No	0.03	0.10
2MS46	21	9.20	6	No	No	No	<0.01	> 0,99
2MS60	26	5.15	7	No	No	No	0.01	> 0,99
2MS67	9	13.60	8	No	No	No	<0.01	0.56
2MS82	20	8.55	7	No	No	No	<0.01	> 0,99
2MS87	11	14.18	18	No	No	No	0.05	> 0,99
2MS91	26	4.39	4	No	No	No	0.01	0.41
Ant11893	22	10.83	9	No	No	No	0.02	> 0,99

Table S4. Estimation of Genotypic linkage disequilibrium for each locus pair across all populations (Fisher's method). Markov chain parameters: Dememorisation: 10000; Batches: 100, Iterations per batch: 5000.

Locus 1	Locus 2	Chi2	df	P-Value	Adj. P-Value (Holm correction)
LX GT 218	2MS87	5.532005	6	0.477597	1.000000
LX GT 218	2MS91	1.057585	2	0.589316	1.000000
2MS87	2MS91	0.510502	2	0.774722	1.000000
LX GT 218	Ant11893	7.247509	4	0.123372	1.000000
2MS87	Ant11893	5.576181	4	0.233114	1.000000
2MS91	Ant11893	0.000000	2	1.000000	1.000000
LX GT 218	Ant3993	1.132616	4	0.889063	1.000000
2MS87	Ant3993	1.276788	4	0.865301	1.000000
2MS91	Ant3993	0.000000	2	1.000000	1.000000
Ant11893	Ant3993	4.419875	4	0.352154	1.000000
LX GT 218	L-18	2.060644	2	0.356892	1.000000
2MS87	L-18	1.745017	2	0.417902	1.000000
2MS91	L-18	0.000000	2	1.000000	1.000000
Ant11893	L-18	0.000000	2	1.000000	1.000000
Ant3993	L-18	0.000000	2	1.000000	1.000000
LX GT 218	LXA GT1	11.704101	4	0.019693	1.000000
2MS87	LXA GT1	3.645658	4	0.456079	1.000000
2MS91	LXA GT1	0.000000	2	1.000000	1.000000
Ant11893	LXA GT1	3.730442	4	0.443714	1.000000
Ant3993	LXA GT1	0.000000	4	1.000000	1.000000
L-18	LXA GT1	0.000000	2	1.000000	1.000000
LX GT 218	2MS17	0.000000	2	1.000000	1.000000
2MS87	2MS17	0.000000	2	1.000000	1.000000
2MS91	2MS17	9.676728	2	0.007920	0.52272
Ant11893	2MS17	0.000000	2	1.000000	1.000000
Ant3993	2MS17	0.000000	2	1.000000	1.000000
L-18	2MS17	0.000000	2	1.000000	1.000000
LXA GT1	2MS17	0.000000	2	1.000000	1.000000
LX GT 218	2MS46	4.211266	4	0.378168	1.000000
2MS87	2MS46	1.818918	4	0.769019	1.000000
2MS91	2MS46	4.357375	2	0.113190	1.000000
Ant11893	2MS46	4.219211	4	0.377151	1.000000
Ant3993	2MS46	1.401730	4	0.843894	1.000000
L-18	2MS46	0.000000	2	1.000000	1.000000
LXA GT1	2MS46	1.213088	4	0.875939	1.000000
2MS17	2MS46	0.000000	2	1.000000	1.000000
LX GT 218	2MS60	0.000000	4	1.000000	1.000000
2MS87	2MS60	0.000000	4	1.000000	1.000000
2MS91	2MS60	0.000000	2	1.000000	1.000000
Ant11893	2MS60	2.616664	4	0.623874	1.000000
Ant3993	2MS60	0.000000	4	1.000000	1.000000
L-18	2MS60	0.000000	2	1.000000	1.000000
LXA GT1	2MS60	0.000000	4	1.000000	1.000000
2MS17	2MS60	0.000000	2	1.000000	1.000000
2MS46	2MS60	0.000000	4	1.000000	1.000000
LX GT 218	2MS67	14.790158	6	0.021953	1.000000
2MS87	2MS67	0.040997	6	0.999999	1.000000
2MS91	2MS67	0.000000	2	1.000000	1.000000
Ant11893	2MS67	5.024634	4	0.284779	1.000000
Ant3993	2MS67	5.845138	4	0.211016	1.000000
L-18	2MS67	0.914412	2	0.633050	1.000000
LXA GT1	2MS67	0.376615	6	0.999033	1.000000
2MS17	2MS67	1.894180	2	0.387868	1.000000
2MS46	2MS67	2.322057	4	0.676756	1.000000
2MS60	2MS67	8.207714	4	0.084259	1.000000

LX GT 218	2MS82	0.919555	4	0.921739	1.000000
2MS87	2MS82	0.339789	4	0.987103	1.000000
2MS91	2MS82	0.000000	2	1.000000	1.000000
Ant11893	2MS82	3.054161	4	0.548803	1.000000
Ant3993	2MS82	0.632147	4	0.959425	1.000000
L-18	2MS82	4.120251	2	0.127438	1.000000
LXA GT1	2MS82	0.000000	2	1.000000	1.000000
2MS17	2MS82	0.858049	2	0.651144	1.000000
2MS46	2MS82	0.000000	4	1.000000	1.000000
2MS60	2MS82	2.137253	4	0.710532	1.000000
2MS67	2MS82	3.156169	4	0.532039	1.000000

Figure S1. Principal Coordinates Analysis (PCoA) generated in GENALEX using Nei's genetic distance matrix. Different symbols are used to indicate different populations. Dark color-coded symbols indicate samples from the different German populations. White color-coded symbols indicate samples from the different other European populations. Large symbols indicate samples collected in 2021, small symbols correspond to sample collected in 2011-2012. Coordinate axis 1 explains 6.15% and coordinate axis 2 explains 4.82% of the variation.

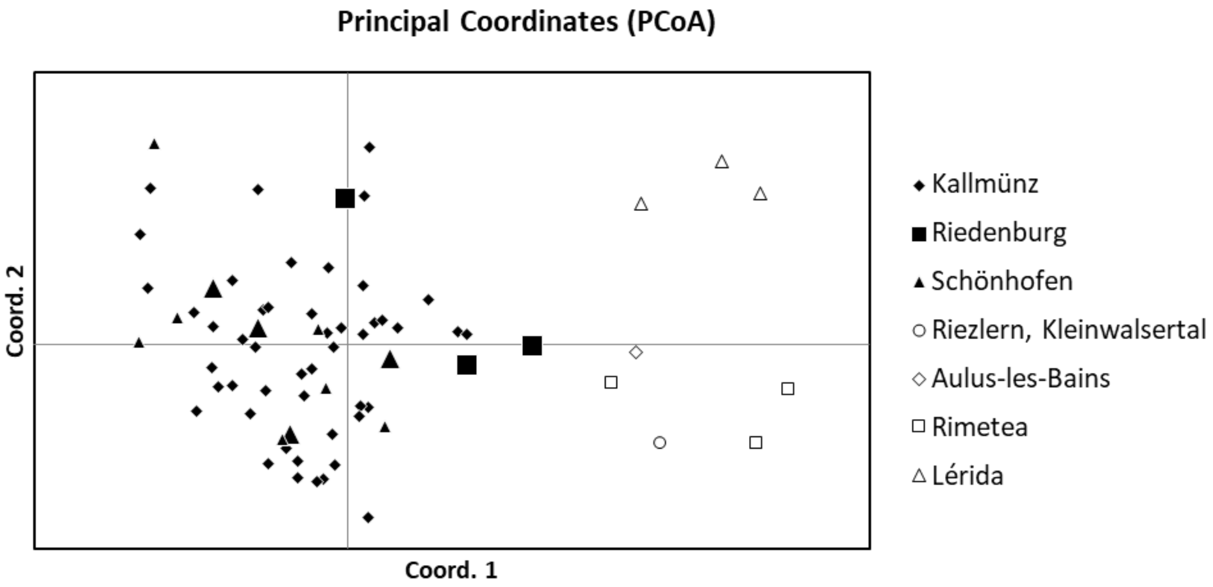

Figure S2. Tree obtained from 49 COI-COII sequences built using the PhyML algorithm with the GTR distance without invariable sites, optimized nucleotide equilibrium frequencies, and tree-searching operations involving best of NNI & SPR. The branch lengths are proportional to estimated divergence along each branch. The color of the sequences' names indicates the geographical origin of the samples. The color of the squares indicates the genotypic cluster of the individuals.

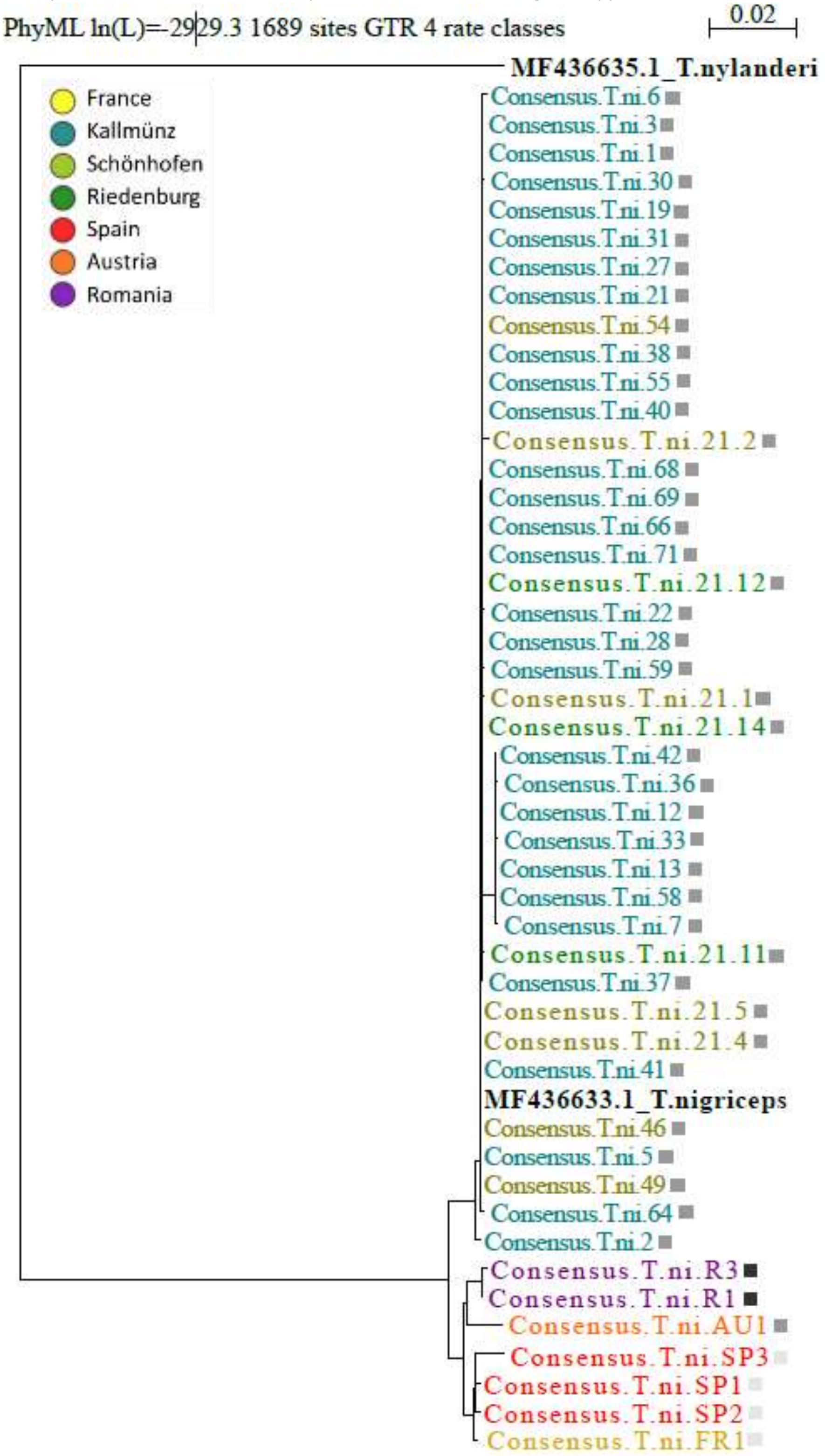

Figure S3. *Left*: Map of the locations of the 30 sequenced colonies of the Kallmünz site. *Right*: haplotypic network based on the mitochondrial sequences. Each disk represents a haplotype. Disk surface is proportional to the number of individuals. The color of the disk indicates the geographical origin of the haplotype. The number of hatches corresponds to the number of mutations between two haplotypes.

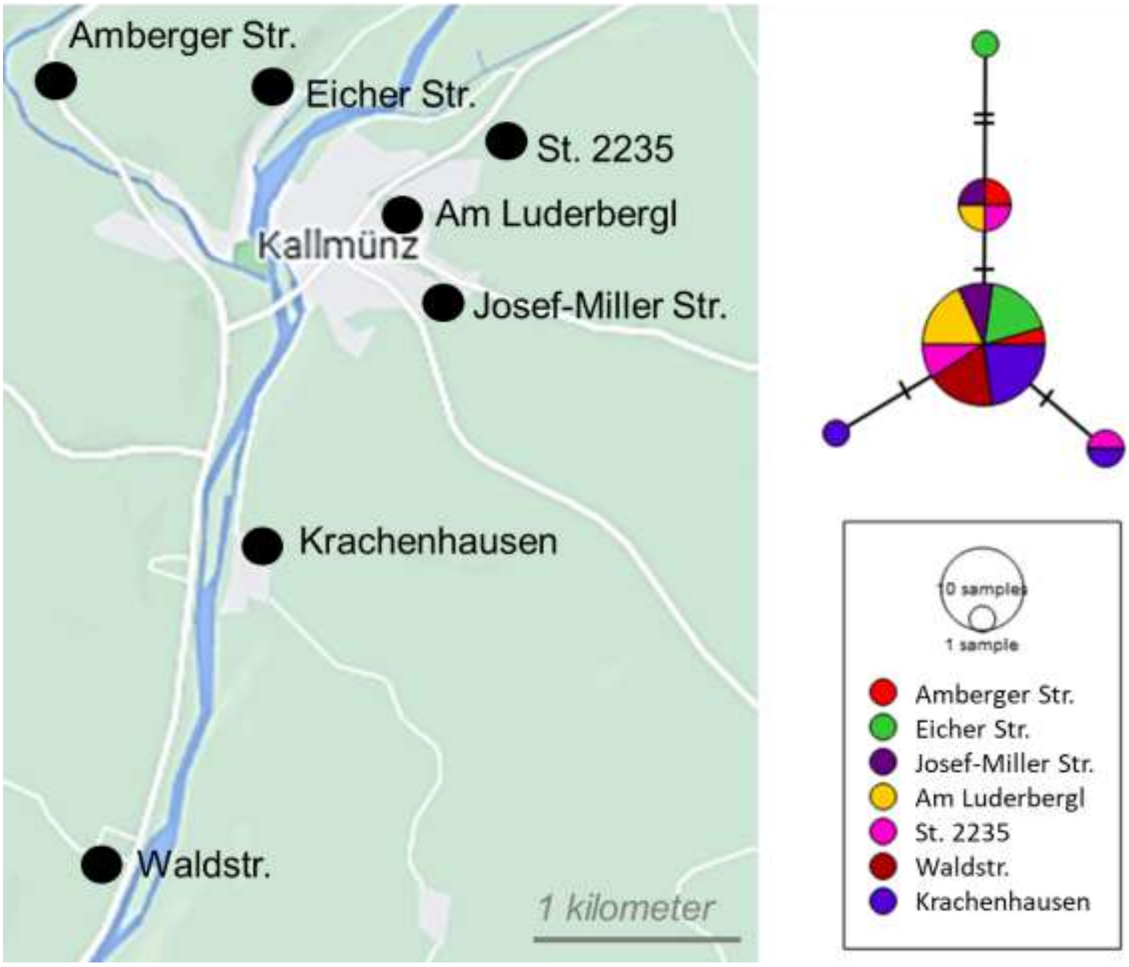

Supplement: Supplementary file 1 — Additional file 1. Additional tables and figures. [file 12862_2022_2044_MOESM1_ESM.pdf]
